# Changes in striatal activity and functional connectivity in a mouse model of Huntington's disease

**DOI:** 10.1371/journal.pone.0184580

**Published:** 2017-09-21

**Authors:** Magali Cabanas, Fares Bassil, Nicole Mons, Maurice Garret, Yoon H. Cho

**Affiliations:** 1 Institut de Neurosciences Cognitives et Intégratives d’Aquitaine, CNRS UMR 5287, Pessac, France; 2 University of Bordeaux, Bordeaux, France; University of Florida, UNITED STATES

## Abstract

Hereditary Huntington’s disease (HD) is associated with progressive motor, cognitive and psychiatric symptoms. A primary consequence of the HD mutation is the preferential loss of medium spiny projection cells with relative sparing of local interneurons in the striatum. In addition, among GABAergic striatal projection cells, indirect pathway cells expressing D2 dopamine receptors are lost earlier than direct pathway cells expressing D1 receptors. To test *in vivo* the functional integrity of direct and indirect pathways as well as interneurons in the striatum of male R6/1 transgenic mice, we assessed their c-Fos expression levels induced by a striatal-dependent cognitive task and compared them with age-matched wild-type littermates. We found a significant increase of c-Fos+ nuclei in the dorsomedial striatum, and this only at 2 months, when our HD mouse model is still pre-motor symptomatic, the increase disappearing with symptom manifestation. Contrary to our expectation, the indirect pathway projection neurons did not undergo any severer changes of c-Fos expression regardless of age in R6/1 mice. We also found a decreased activation of interneurons that express parvalbumin in the dorsomedial striatum at both presymptomatic and symptomatic ages. Finally, analysis of c-Fos expression in extended brain regions involved in the cognitive learning used in our study, demonstrates, throughout ages studied, changes in the functional connectivity between regions in the transgenic mice. Further analysis of the cellular and molecular changes underlying the transient striatal hyperactivity in the HD mice may help to understand the mechanisms involved in the disease onset.

## Introduction

Huntington’s disease (HD) is a hereditary neurodegenerative disease caused by a polyglutamine expansion in the gene product, Huntingtin. The mutant protein is known to form nuclear inclusions that impair cell functions and produce cell death. HD is associated with not only its hallmark chorea and other motor impairments, but also its cognitive and psychiatric disturbances; the manifestation of the latter often precedes the motor symptoms [1].

Although huntingtin protein is ubiquitously expressed, a major alteration is observed in the striatum, main output structure of the basal ganglia [2–4]. GABAergic medium spiny neurons (MSNs) are a major constituent of the striatum, and are divided into two sub-populations without anatomical segregation: one expressing mainly dopamine D1 receptor (D1R), and the other expressing dopamine D2 receptor (D2R). These two subpopulations of projection cells are the origins of the direct striatonigral and indirect striatopallidal pathways, respectively, and are both regulated by different classes of local interneurons (INs). Studies on *postmortem* HD patient brains have shown that indirect pathway MSNs in the striatum are affected earlier than direct pathway MSNs [5–7]. However, all neuronal types including direct pathway MSNs and local INs also are affected at late stages of the disease [8–11].

While *in vitro* studies on mouse models of HD have suggested a compromised functional integrity of striatal microcircuit [12–14], there exist few, if any, studies examining its alterations *in vivo*. To address this question, we and others have conducted extracellular single unit electrophysiological recordings in freely behaving HD transgenic mice [15–16]. In particular, we recorded early symptomatic R6/1 mice during an operant conditioning task that relies critically on the integrity of the striatum. During random sampling of cells in the dorsal striatum only a small percent of cells recorded were MSNs [15]. Indeed, the majority of the recorded cell types in these HD mice were qualified as fast-spiking INs in contrast to 50% being INs in WT mice. These data suggest that MSNs may suffer from functional dysregulation *in vivo* in R6/1 mice. Because extracellular single-unit recording technique did not allow dissociation between the sub-populations of direct and indirect pathway MSNs, a hypothesis was formulated that a few recorded MSNs in HD mice might belong to the direct pathway that may remain functional, and “unrecordable” MSNs to be part of the indirect pathway.

Here, we employed semi-quantitative analysis of striatal activity by assessing the c-Fos protein expression (as a surrogate marker of neuronal activity *in vivo*) [17–19] induced by cognitive and behavioral stimulation in R6/1 mice of different disease states. In addition to the striatum, we also assessed c-Fos expression in multiple brain regions to potential alteration of functional connectivity between these regions of interest.

## Materials and methods

### Animals

Experiments were performed on male wild-type (WT) (n = 41) and R6/1 transgenic littermate (n = 46) mice of 3 different ages: 2, 4 and 6 months representing, respectively, pre- symptomatic, early motor symptomatic and late symptomatic stages (Table 1). The mice were bred in the animal facility of Bordeaux University and were obtained from a parent genitor R6/1 mouse (B6.Cg-Tg(HDexon1)61Gpb/J, Stock number: 006471, Jackson Laboratory, Main Harbor, NY, USA) crossed with female C57BL/6 mice (IFFA/Credo, Lyon, France). The R6/1 line expresses exon 1 of the human HD gene with an expanded number of CAG trinucleotide repeats (123.64 ± 0.89). Genotypes were tested by PCR of tail biopsy specimens. All animals were housed in polycarbonate standard cages (33 x 15 x 14 cm in size; Tecniplast, Limonest, France), provided with sawdust bedding (SAFE, Augy, France) and a stainless steel wire lid. Food chow (SAFE, Augy, France) and water were provided *ad libitum* until experiment began. The animals were maintained in a colony room under temperature (22°C) and humidity-controlled (55%) conditions with a 12:12 hr light–dark cycle (lights on at 7 a.m.).

**Table 1:** Experimental plan used.

	R6/1		Wild-type		Total
Age (disease status)	Learning	Control	Learning	Control	
2 months (pre-symptomatic)	9	9	9	7	34
4 months (early symptomatic)	8	6	8	5	27
6 months (late symptomatic)	9	5	7	5	26
Total	26	20	24	17	87

The number of mice used for two genotypes (WT and R6/1), two experimental conditions (Learning and home cage control), and three ages/disease statuses (2, 4 and 6 months). R6/1 mice are considered to be pre-motor symptomatic at 2 months, early motor-symptomatic at 4 months and fully symptomatic at 6 months of age. Brains of only a subgroup of animals (grey shaded) were processed for multiple labeling Immunofluorescence experiments.

### Behavioral test

#### Apparatus

**Fig 1:**
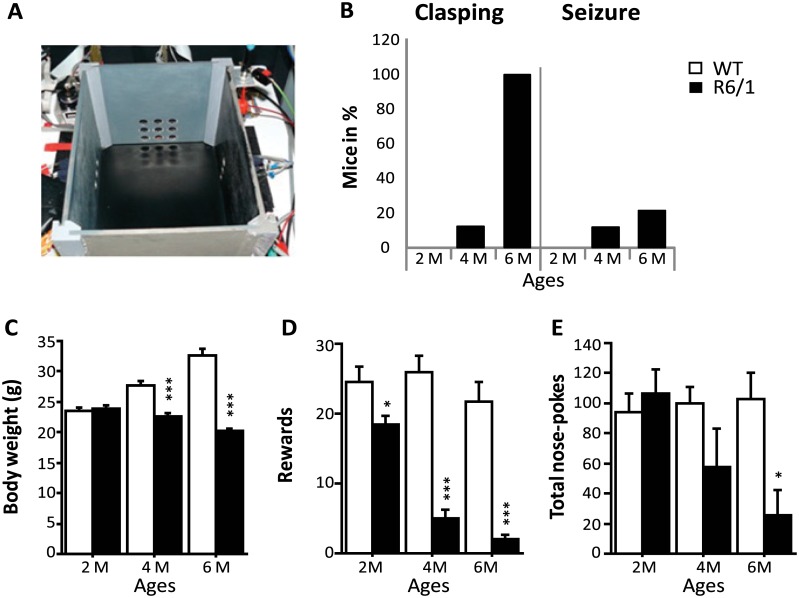
Behavioral performance in 2-, 4- and 6-month-old WT and R6/1 mice. A. Apparatus used for operant conditioning in learning groups. B. % mice displaying clasping and seizure. C. Body weight changes across ages. D-E. The number of rewards received and exploratory activity (number of total nose-poke responses) during the single 30 min session of operant conditiong.

The apparatus used was a rectangular operant chamber (28 x 18 x 18cm) constructed of grey PVC (Fig 1A). The chamber was equipped with 11 nose-poke holes (13 mm in diameter) placed over three of its inner walls [15]. Each nose-poke hole is equipped with an illuminating light emitting diode. Photocells placed behind the nose-poke holes were used for the detection of the mouse nose poking into the holes. On the floor of the fourth wall was located a liquid dispenser delivering 7 μl of sweetened whole milk as reinforcement through a catheter via an electro-valve release mechanism.

#### Behavioral protocol

Animals of 3 different ages were randomly assigned to either “Learning” or “home cage control” group and were housed individually for a week. They were then food deprived progressively to lose 10%–15% of their weight over a 4 day period prior to testing day. During this period, mice were handled daily and habituated to sweetened whole milk (2% sugar) to be used as a reward. Mice of each age and genotype were then subdivided into “learning” and “home cage control” groups. During the testing day, mice of learning groups (n = 50) were habituated for 30 minutes in the testing room and then placed in the operant chamber for additional 30 minutes. The mice learned to associate the arrival of reward with a nose poking into any of the 11 holes placed in the chamber. Only first nose responses following reward consumption were considered as correct responses. Therefore, nose-poke responses made in the interval between the first correct response and the food consumption were recorded as incorrect responses. Healthy C57BL/6 mice require the task in general 5–10 daily sessions of 30 minutes, while R6/1 mice necessitate three times more to reach asymptote level, which in addition, remains lower than in WT mice [15]. The number of correct and total (correct + incorrect) nose-poke responses were collected and analyzed, the total responses were considered as a measure of the general level of exploratory activity.

Following the completion of one single session of the 30 minutes behavioral test in which the testing order of different genotype groups was counterbalanced, mice were placed in their home cages and returned to the animal colony room. They were then left undisturbed for 1 hour before sacrifice by perfusion. Mice of home cage control groups were kept in their home cages until the sacrifice, while they received the same procedure as mice of learning groups for food deprivation, individual housing and handling. Experiments were conducted in the morning. Behavioral analysis was performed by an experimenter blind to genotype and age.

#### Weight, clasping and seizure

All mice were weighed daily during food deprivation and test periods. The clasping test [20] was used to identify the motor phenotype. Clasping is characterized by the abnormal crossing or joining of the hind with the fore paws. During the handling and manipulation, overt behavioral seizures also were monitored [21]. Clasping and seizure phenotypes were coded in a binary manner (present or absent).

### Immunocytochemistry

#### Preparation of brains

Mice were deeply anesthetized with an overdose of sodium pentobarbital (150 mg/kg, ip) and transcardiacally perfused with 4% paraformaldehyde solution. Brains were stored overnight in the same fixative solution and then transferred to 30% sucrose solution for an additional 72 hours before being cut into 50-μm thick coronal sections on a freezing microtome (Leica, Mannheim, Germany). Sections were kept at -20°C in a cryoprotectant solution [30% glycerol (v/v) and 30% ethylene glycol (v/v) in 0.1M phosphate buffer (PB, pH: 7.4)] until processed for immunohistochemistry or immunofluorescence.

#### Single-labeling c-Fos immunochemistry

Free-floating sections were first rinsed in phosphate buffered saline (PBS, pH: 7.4) and then treated 10 minutes with 1% H_2_O_2_ in 10% methanol to inhibit endogenous peroxidases followed by an incubation for 1 hour in a blocking solution containing 0.2% Triton-X 100 and 5% normal goat serum. They were then rinsed in PBS before being incubated for 48 hours at 4°C with anti-c-Fos rabbit polyclonal antibody (1:5000; Santa Cruz Biotechnology, Santa Cruz, CA), then with biotinylated goat anti-rabbit IgG (1:2000; Jackson Immunoresearch) followed by an avidin-biotin-peroxidase complex (Vectastain ABC Elite kit, Vector Laboratories, Burlingame, CA). The peroxidase activity was visualized in Tris solution containing 0.025% diaminobenzidine and 0.03% H_2_O_2_. Finally, sections were mounted on gelatin-coated glass slides, dehydrated through a graded series of ethanol, transferred to toluene, and then cover slipped with the Eukitt mounting medium.

Images were acquired using an Olympus (BX50) through a 20x objective and digitized using an imaging analysis system (Biocom Visiolab 2000, V4.50). To calculate the mean number of c-Fos positive (c-Fos+) nuclei/mm^2^ per region of interest, 6–8 independent measures (from separate sections and hemispheres) were taken and averaged for each brain. Cell counts were made in several regions based on the mouse brain stereotaxic atlas (Paxinos and Franklin, 2013). The sections of group to be directly compared were processed at the same time and using the same conditions and reagents in order to reduce variability. All analyses were performed by an experimenter blind to the genotype, age and experimental conditions.

#### Multiple-labeling by immunofluorescence in the dorsomedial striatum

Coronal sections containing dorsal striatum were first incubated for 1 hour in blocking PBS solution containing 4% donkey serum and 0.3% Triton X-100 (Sigma, St. Quentin Fallavier, France). They were then incubated for 72 hours at 4°C with primary antibodies (see Table 2) diluted with the blocking solution. They were then rinsed in PBS and incubated for 1 hour in blocking solution containing a cocktail of secondary antibodies conjugated to fluorescent probes (Alexa-488, -568, or -647-conjugated donkey anti-mouse, anti-rabbit, anti-guinea pig or anti-goat antibody; Jackson Immunoresearch). Sections were washed, mounted on glass slides and cover slipped. Observation and acquisition were performed with a Leica DM6000B microscope (Leica Mannheim, Germany) equipped with a Qimaging RETIGA.

**Table 2:** Antibodies used in immunofluorescence labeling.

Antibody	Host	Dilution	Company	Reference number
c-Fos	Rabbit	1: 3000	Santa Cruz Biotechnology	Sc-52
DARPP-32	Mouse	1: 2000	BD Biosciences	611520
D1R	Goat	1: 4000	Frontier Institute	Af1000
D2R	Guinea pig	1: 4000	Frontier Institute	Af500
PV	Guinea pig	1: 4000	Synaptic System	195004
ChAT	Goat	1: 400	Millipore	AB144P
nNOS	Guinea pig	1: 4000	Frontier Institute	Af740
Calr	Mouse	1: 2000	Synaptic System	214 111

Abbreviations: DARPP-32, dopamine- and cyclic-AMP-regulated phosphoprotein of molecular weight 32,000; D1R, Dopamine D1 receptor; D2R, Dopamine D2 receptor; PV, Parvalbumin; ChAT, Acetylcholinesterase; nNOS, Neuronal nitric oxide synthase; Calr, Calretinin.

To identify c-Fos+ nuclei in D1R- or D2R-expressing MSNs, quadruple-labeling immunohistochemistry was performed using antibodies against D1R, D2R, DARPP-32 in addition to c-Fos. Image stacks (5 stacks with a step of 2 μ) within the dorsomedial striatum (DMS) at 1.2 mm– 0.7 mm anterior to Bregma level (Paxinos, and Franklin, 2004) were acquired using Micromanager to control the acquisition process with an oil immersion 40X lens.

Each measure was collected on 7 randomly chosen images of the DMS (5 stacks with a step of 2 μm) and 6 independent measures (from separate sections and hemispheres) were taken and averaged for each mouse brain. All cells expressing c-Fos and DARPP-32 were tagged using ImageJ multi-point tool and considered as c-Fos+ MSNs. To identify each c-Fos+ MSN as either D1R+ or D2R+ cell, the outline of the cell was drawn according to the DARPP-32 labeling that filled the cell. Then, the fluorescence ratio of D1R labeling on the cell periphery versus D1R labeling of the whole image was measured followed by the same process for D2R labeling; cell type was identified by the higher D1R or D2R signal-to-background ratio at the cell periphery. All analyses were performed by an experimenter blind to genotype and age. Data were expressed as percent of D1R and D2R expressing cells among c-Fos+ MSNs.

Double labeling immunofluorescence experiments were performed to identify c-Fos+ INs. Striatal sections were incubated with anti-c-Fos antibody and either anti-acetylcholinesterase (ChAT), anti-parvalbumin (PV), anti-neuronal nitric oxide synthase (nNOS) or anti-calretinin (Calr) antibody according to the procedure described above. For each DMS, stacks of 5 images with a step of 5 μm were taken using Micromanager for acquisition as previously. A 20X lens was used to take mosaic image stacks of the DMS, and striatal sections were analyzed as above. All stained INs revealed by IN-specific antibodies were tagged using ImageJ multi-point tool and the number of c-Fos+ cells among each IN subtype was counted. All analyses were performed blind to genotype and age. Data were expressed as percent of c-Fos+ cells among IN subtypes.

#### Experimental design and statistical analysis

Experimental plan involved 41 WT mice and 46 R6/1 mice of 3 different ages: 2, 4 and 6 months (see Table 1). Each age and genotype group was randomly subdivided into two experimental conditions: learning vs home cage control. Behavioral and immunocytochemical data from these animals were analyzed using multivariate analysis of variance by considering age (2, 4 and 6 months), genotype (WT and R6/1), and experimental conditions (learning vs home cage control) as between-group factors. Student Newman-Keuls post-hoc test was used if necessary with significance set at p<0.05. Data that were not normally distributed were analyzed using nonparametric Mahn-Whitney test. Data were presented as mean ± SEM.

Correlation (Spearman r) matrix was calculated for c-Fos+ cell counts obtained from all brain regions of interest (as well as behavioral measures for mice from learning groups) to assess functional connectivity during learning and home cage control conditions. Statview (SAS Institute Inc, version 5.0.1) was used for statistical analyses.

## Results

### Weight, clasping and seizures

Body weight, clasping and seizure occurrence were monitored to confirm disease statuses of our R6/1 mice of different ages studied [22]. Fig 1 shows the age-associated differences between WT and R6/1 mice. First, 12.5% of 4 month-old and 100% of 6 month-old R6/1 mice displayed clasping (Fig 1B). The seizure was also observed in 12.5% and 22.2% of R6/1 mice of 4 and 6 months of age, respectively (Fig 1B). In contrast, none of the 2-month-old R6/1 or WT mice of any age exhibited these phenotypes.

There was no significant difference in weight between the two genotype groups at 2 months of age (Fig 1C). Then, R6/1 mice stopped gaining weight as of 4 months, while WT mice continued to do so with further ages (age x genotype interaction: F(2, 79) = 52.707; p<0.0001). Significant differences in weight were observed between WT and R6/1 mice at both 4 and 6 months (both p<0.05), but not 2 months (Fig 1C).

### Operant conditioning

We recently showed an alteration, in vivo, of MSN activity in 4 month-old R6/1 mice during an operant conditioning task [15]. We, therefore, submitted our mice to this task to induce c-Fos expression. R6/1 mice, as compared to WT mice, in general, obtained the lower amount of rewards (following correct nose-poke responses) during the 30 min test session (genotype effect: F(1,38) = 93.379; p<0.0001), and the significant differences were observed at all ages studied (all p<0.05). Additionally, the amount of rewards received declined further across the three age groups of R6/1 mice (age x genotype interaction: F(2,38) = 8.42; p = 0.007) (Fig 1D). The numbers of total nose-poke responses in R6/1 mice also declined with age but were significantly lower than in WT mice only at 6 months (p<0.05), but not 2 and 4 months (p>.05) (genotype x age interaction: F(2,38) = 3.91; p = 0.028, Fig 1E). Finally, these behavioral measures were not significantly correlated with body weight when analysis was performed for each age and genotype group.

### Region-specific patterns of Fos-immunoreactivity associated with learning

**Fig 2:**
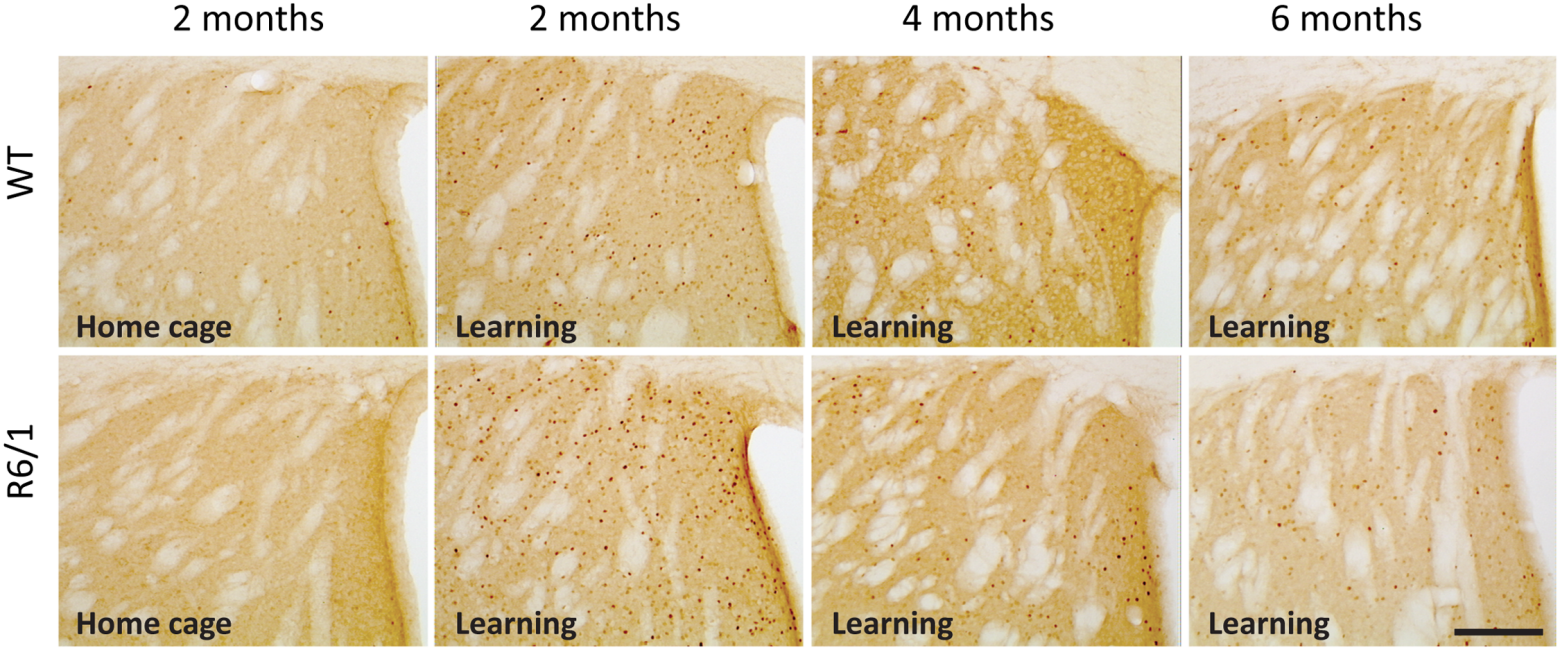
Comparative and representative images of c-Fos immunostaining in coronal sections of the dorsomedial striatum in R6/1 and WT mice of 3 age groups from learning and 2 month home cage control conditions. Scale bar, 100 μm.

To analyze the impact of learning on neuronal activation, c-Fos expression was assessed on several brain regions. More precisely, c-Fos+ cell counts were performed in not only the DMS and dorsolateral striatum (DLS), but also regions interconnected with the striatum important for and/or usually activated by cognitive stimulation such as the prefrontal cortex, septum, CA1 and dentate gyrus (DG) of the dorsal hippocampus, and lateral nucleus of amygdala. Representative c-Fos immumnostainings in the DMS are shown in Fig 2. The lateral nucleus of the amygdala was included in the analysis because its activation may reveal unspecific and stress-related activation in the other regions of interest.

In home cage animals, there existed no significant genotype effects in all structures except for the DMS in which c-Fos+ cell counts were significantly lower in R6/1 mice as compared to WT littermates (genotype effect: F(1,31) = 8.00, p<0.0081) (data not shown). Furthermore, c-Fos+ cell counts increased with age, irrespective of genotype, in the DG of the hippocampus (age effect: F(1,31) = 4.78, p = 0.015) and septum (age effect: F(1,31) = 6.62, p = 0.004) (data not shown).

As compared to home cage control groups, the number of c-Fos+ cells in learning groups was significantly higher whatever the regions examined (p<0.0001 for all regions) irrespective of age and genotype.

**Fig 3:**
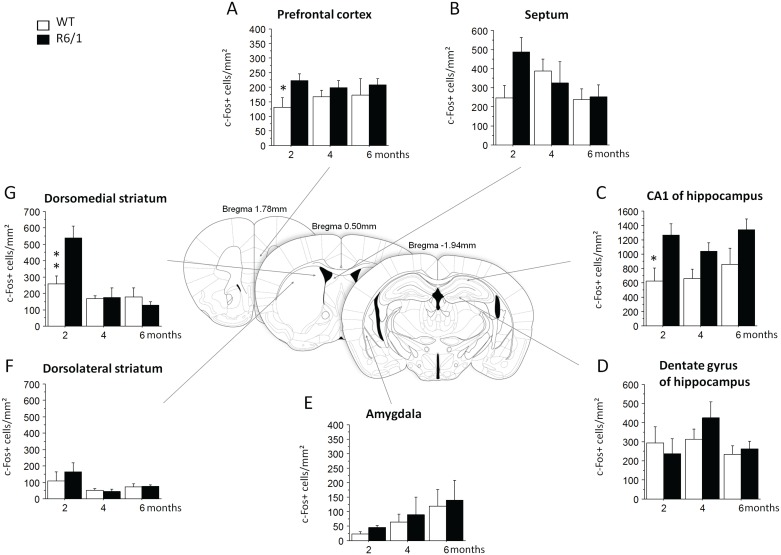
c-Fos positive cell counts in WT and R6/1 mice of learning groups. Operant conditioning-induced c-Fos expression in the prefrontal cortex, dorsomedial striatum, dorsolateral striatum, septum, CA1 and dentate gyrus of the hippocampus, and lateral nucleus of amygdala. * significant difference (p < .05) between age-matched genotype groups. 2 months: WT, n = 8, R6/1, n = 7; 4 months: WT, n = 8, R6/1, n = 6; 6 months: WT, n = 7, R6/1, n = 8.

In learning groups, contrary to our expectation of deficient striatal activation associated with HD mutation, c-Fos-expressing cell counts in the DMS were, in general, greater in R6/1 mice (genotype effect: F1,38) = 4.11, p = 0.0497), and more particularly at pre-symptomatic 2 months of age (p<0.01, Fig 3G). No such genotype-related difference was found any more at later 4 and 6 months motor symptomatic ages (age x genotype interaction: (F2,38) = 7.02, p = 0.0025). Contrary to the DMS, no significant difference between the genotypes was found for the DLS (Fig 3F).

Similar age-associated changes, in R6/1 mice, of c-Fos expression also were observed in the prefrontal cortex (age x genotype interaction: F(2,38) = 3.92, p = 0.055, Fig 3A), with significantly higher c-Fos+ cell counts as compared to age-matched WT mice at 2 months (p<0.05), but not later ages. In addition, c-Fos+ cell counts in R6/1 mice, regardless of age, were significantly increased in the dorsal CA1 field of the hippocampus (genotype effect: F(1,38) = 13.12, p = 0.0008, Fig 3C), but the increase was significant only at 2 months (<0.05). No significant changes were found in the DG of the hippocampus (Fig 3D), septum (Fig 3B) and lateral amygdala (Fig 3E, Table 3).

**Table 3:** Changes of c-Fos+ cell counts in different regions of the limbic circuit in R6/1 mice.

Anatomical regions	2 months	4 months
Dorsomedial striatum	↑	=
Dorsolateral striatum	=	=
Prefrontal cortex	↑	=
CA1 of the hippocampus	↑	=
Amygdala, DG, Septum	=	=

↑, increase; =, no change of activity; DG, dentate gyrus of the hippocampus.

### Changes of functional connectivity in both home cage control and learning groups

**Fig 4:**
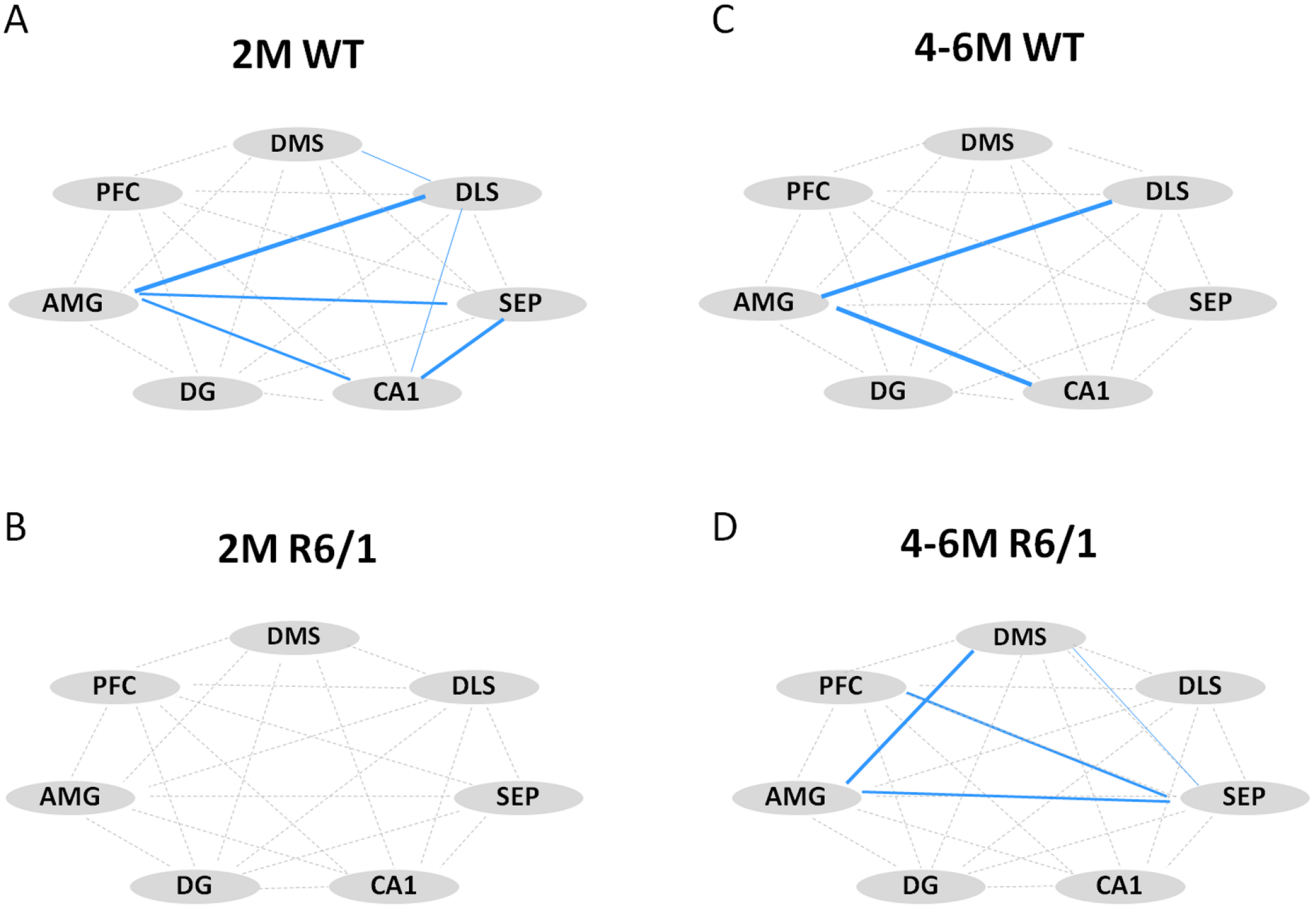
Correlative activity of different regions of interest in home cage control groups of 2 month-old (WT, n = 7; R6/1, n = 9) and 4–6 month-old (WT, n = 10; R6/1, n = 10) mice. Positive Spearman rho correlations are shown in blue. Thickness of line is proportional to statistical significance (0.07>p>0.0001). DMS: dorsomedial striatum, DLS: dorsolateral striatum, SEP: septum, AMG: lateral nucleus of amygdala, PFC: prefrontal cortex, DG: dentate gyrus of the hippocampus.

**Fig 5:**
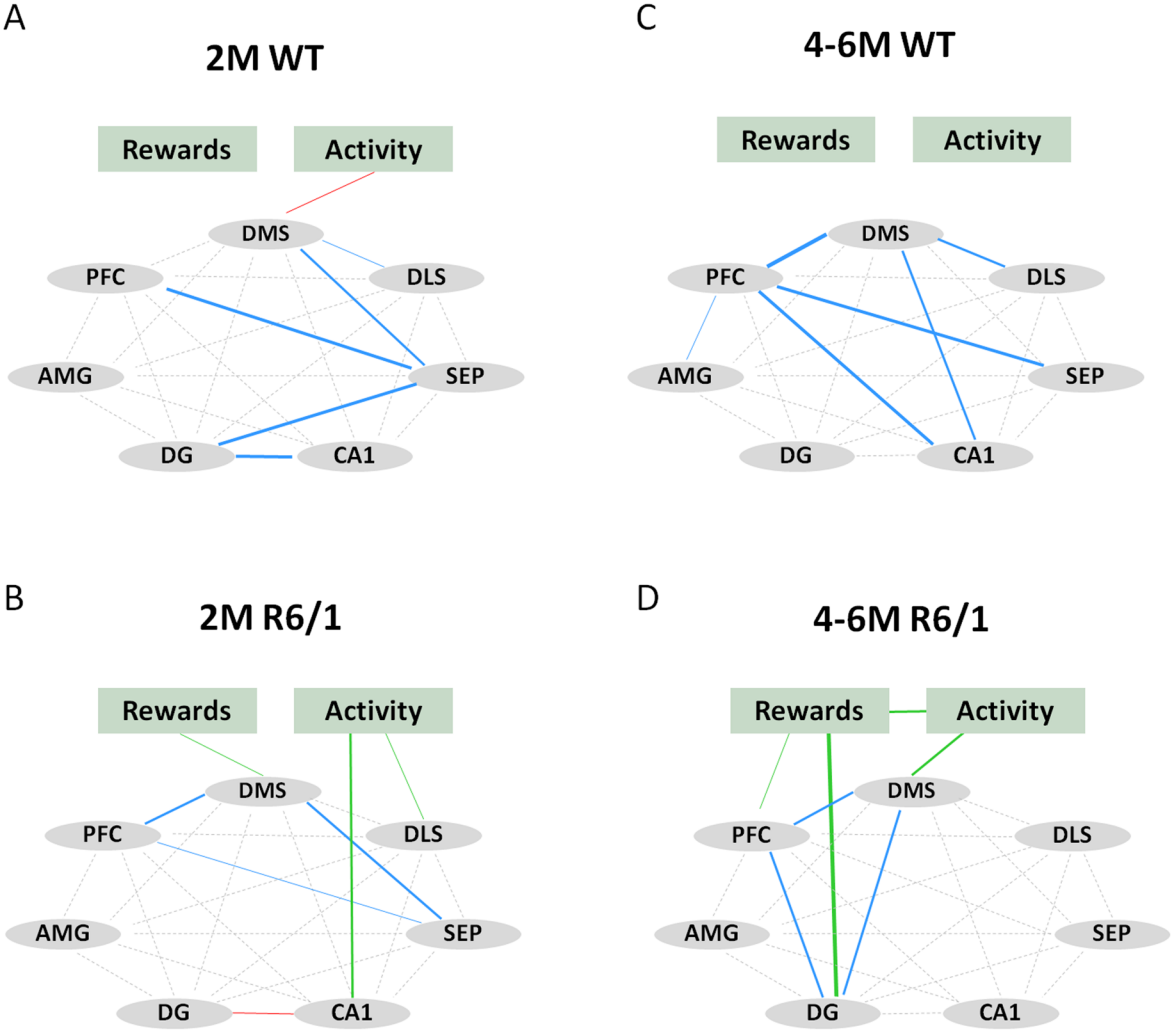
Correlative activity of different regions of interest and behaviors (rewards and activity) in learning groups of 2 month-old (WT, n = 8; R6/1, n = 7) and 4–6 month-old (WT, n = 15; R6/1, n = 14) mice. Positive correlations between different regions of interest are shown in blue. Positive correlations between c-Fos labeling and behaviors are shown in green, and negative correlations in red. Thickness of lines is proportional to statistical significance (0.07>p>0.0001). DMS: dorsomedial striatum, DLS: dorsolateral striatum, SEP: septum, AMG: lateral nucleus of amygdala, PFC: prefrontal cortex, DG: dentate gyrus, Rewards: number of rewards received, Activity: number of total nose-pokes.

We then analyzed functional connectivity among brain regions of interest examined by calculating correlation matrix of their c-Fos+ counts in both home cage control and learning conditions (Figs 4 and 5). Here we combined data from 4- and 6-month-old mice for each genotype due to the similarity data between these two ages. Correlation analysis showed that home cage WT mice displayed strong correlations between the amygdala and several regions of interest at both ages studied (Fig 4A and 4C). To the contrary, such correlations were absent in 2 month-old R6/1 mice (Fig 4B), while rather extended regions were co-activated in 4–6 month-old R6/1 mice (Fig 4D).

Learning induced, in WT mice, the co-activation of the DMS, DLS, prefrontal cortex, septum and hippocampal subregions (Fig 5A and 5C), even though the patterns of activation remained quite different for the two ages considered. To the contrary, in R6/1 mice learning resulted in inter-related activity only among restricted regions regardless of age considered (Fig 5B and 5D). The limited circuit concerned the DMS, prefrontal cortex and septum at 2 months, and the DMS, prefrontal cortex and DG of the hippocampus at 4–6 months. In addition, c-Fos+ counts in these regions were significantly and positively correlated with behavioral performances (i.e. rewards and total nose-poke responses) in R6/1 mice, relationships not seen in WT mice of any age.

### D2R- as well as D1R-expressing MSNs were recruited normally in R6/1 mice

**Fig 6:**
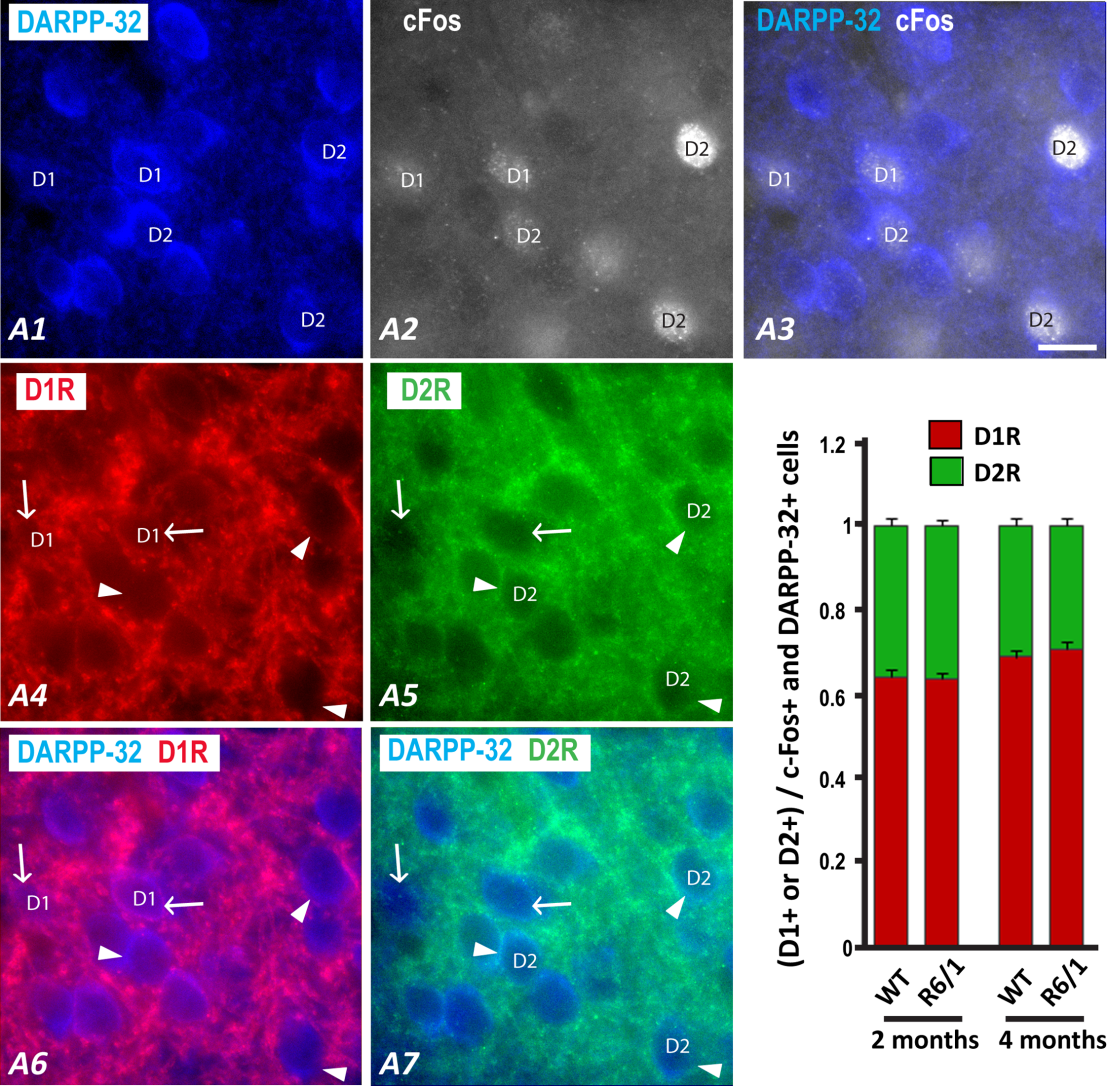
Fluorescent multiplelabeling of neurons expressing DARPP-32, D1R, D2R, and c-Fos in coronal sections of the dorsomedial striatum in 2 month-old (WT, n = 9; R6/1, n = 9) and 4 month-old (WT, n = 8; R6/1, n = 7) mice. A coronal section of the dorsal striatum was labeled for DARPP-32 (A1), c-Fos (A2), D1R (A4), and D2R (A5). Merged images (A3) illustrate that c-Fos is detected in the nucleus of a number of DARPP-32+ cells. Merged images (A6, A7) illustrate that double-labeled DARPP-32/c-Fos neurons are identified as either D1R (A6, arrows) or D2R (A7, arrowheads) positive neurons according to the labeling with the corresponding antibody. Scale bar, 10 μm. B) The histogram shows the ratio of D1R (red) or D2R (green) expressing cells among cells labeled by both DARPP-32 and c-Fos antibodies (C-Fos+ MSNs).

To assess whether c-Fos expressions in distinct MSN populations (expressing D1R *vs* D2R) in the striatum are differentially altered in R6/1 mice, we performed multiple immunofluorescence experiments. Striatal sections from 2 and 4-month learning groups were processed for quadruple-immunofluorescence to segregate, within c-Fos+/DARPP-32+ MSN population, D1R+ from D2R+ cells (Fig 6A). This analysis was centered on the DMS for its critical involvement in the early acquisition of a procedural task [23]. Since R6/1 mice are characterized by the decreased DARPP-32 immunoreactivity in the striatum at late symptomatic ages [24–25], we did not process brain sections from 6 month-old mice to prevent any false-negative labeling of MSNs.

Across ages and genotypes, we found more D1R+ (66% ± 0.05) than D2R+ (34% ± 0.05) cells within c-Fos+/DARPP32+ cells (receptor type effect: F(1,29) = 374.64, p<0.0001, Fig 6B). Surprisingly, this proportion was not different between WT and R6/1 mice across two ages (genotype effect: F(1,29)<1, n.s., genotype x receptor type interaction: F(1,29)<1, n.s.). In addition, the proportion of D1R+ cells among c-Fos+/MSNs increased significantly with age irrespective of the genotype, while that of D2R+ cells accordingly decreased (receptor type x age interaction: F(1,29) = 9.15 p<0.01). Interestingly, we also found some c-Fos expression in cells that did not show apparent DARPP-32 labeling, suggesting that a number of c-Fos+ cells might be INs.

### Early functional alterations of PV-expressing INs in R6/1 mice

**Fig 7:**
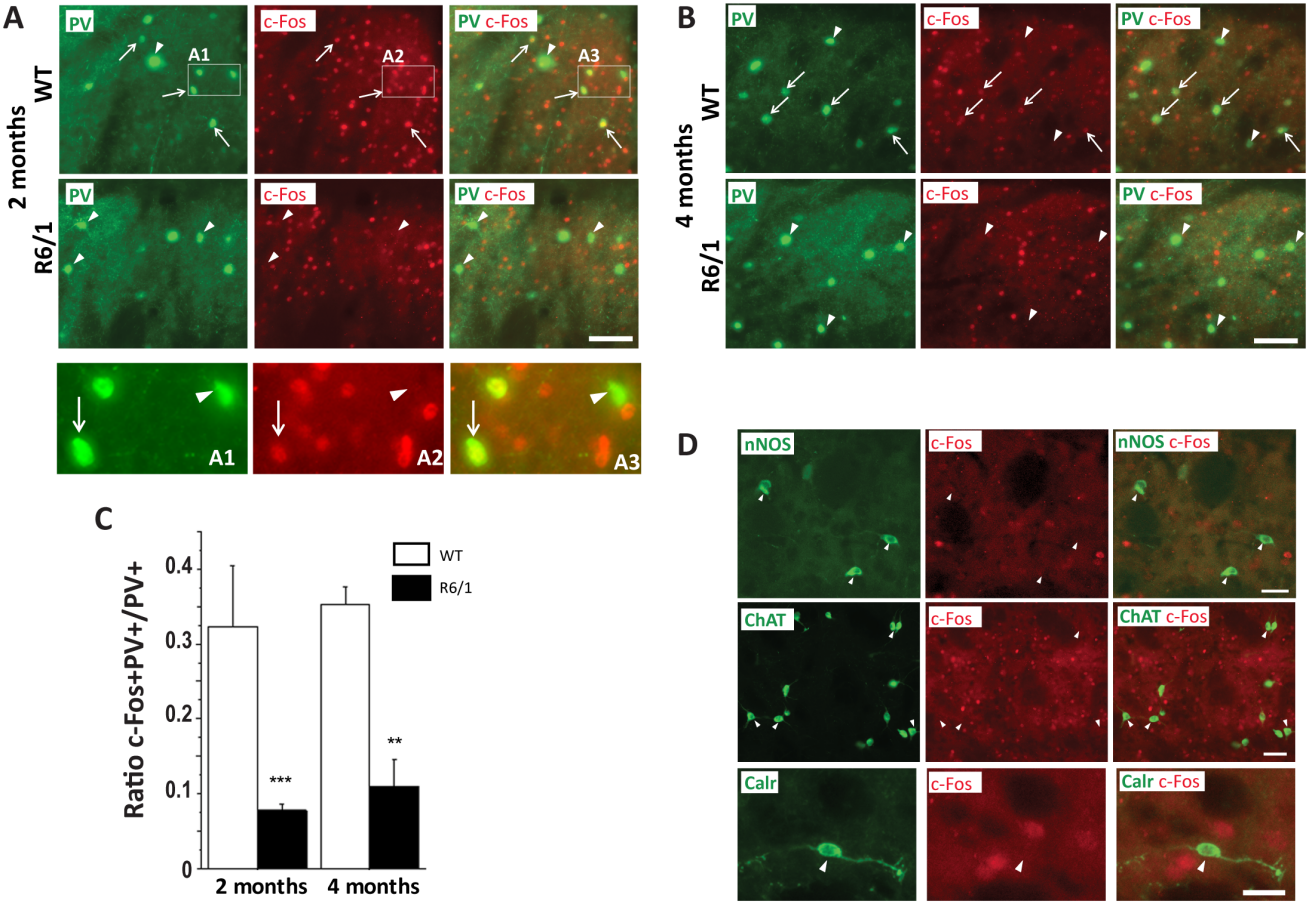
Fluorescent multiplelabeling of INs expressing c-Fos in coronal sections of the dorsomedial striatum in 2 and 4 month-old R6/1 (n = 4 for both ages) and WT (n = 4 for both ages) mice. A. B. Representative images showing fluorescent labeling for parvalbumin (PV) and c-Fos in the striatum of WT or R6/1 mice at 2 and 4 months as indicated on the left (white frames in A detailed in A1-3). c-Fos labeling is detected (arrow) or not detected (arrowhead) in the nucleus of PV+ cell bodies. Scale bar, 50 μm. C. Ratio of PV neurons expressing c-Fos among total PV+ neurons. D. Representative images in the striatum of 4 month-old WT mice showing that c-Fos labeling is not detected (arrowhead) in the nucleus of INs expressing nitric oxide synthase (nNOS, Scale bar, 20 μm), acetylcholinesterase (ChAT, Scale bar, 100 μm), or calretinin (Calr, Scale bar, 10 μm). **p< 0.01; ***p<0.001 significant difference between age-matched genotype groups.

To determine potential changes of striatal IN activity in R6/1 mice, we performed a dual immunofluorescence labeling study (Fig 7, Table 4). Fast-spiking INs are characterized by their expression of PV, while PLTS (persistent depolarizing plateau low threshold Ca^++^ spike) INs are characterized by their expression of nNOS [26–27]. Other major GABAergic INs express Calr, while cholinergic INs express ChAT [27]. We, therefore, performed multilabelimmunostaining on striatal sections from 2- and 4-month learning groups; in addition to c-Fos, we labeled PV-, ChAT-, nNOS-, or Calr-expression (Fig 7A, 7B and 7D). We were able to quantify c-Fos labeling in PV+ neurons in the DMS of both genotypes in 2 and 4 month-old mice (Fig 7A and 7B). Interestingly, Mahn-Whitney analyses showed that the proportion of c-Fos+ cells among this class of INs was significantly smaller in R6/1 mice (genotype effect: U = 1, U’ = 63.00, p = 0.0011, Fig 7C), this at both ages studied (p<0.05 for 2 and 4 months). Only negligible proportions (<1%) were c-Fos+ among Calr-, ChAT-, and nNOS-expressing INs such that no further analysis could have been performed (Fig 7D, Table 4).

**Table 4:** Changes of c-Fos+ cell proportions in different cell types in the dorsomedial striatum of R6/1 mice at 2 and 4 months of age.

Proportion of cell types in the dorsomedial striatum	HD vs WT 2 months	HD vs WT 4 months
D1R+/DARPP-32+ and c-Fos+	=	=
D2R+/DARPP-32+ and c-Fos+	=	=
c-Fos+/PV+	↓	↓
c-Fos+/ChAT+, /Calr+, /nNOS+	= (0)	= (0)

Data are shown as the proportion, among c-Fos+ MSNs (DARPP-32+|c-Fos+) of direct pathway MSN expressing Dopamine D1R and indirect pathway MSNs which express Dopamine D2R. Percent of c-Fos+ nuclei in 4 classes of INs, which express parvalbumin (PV), acetycholinesterase (ChAT), calretinin (Calr) and nitric oxide synthase (nNOS).

## Discussion

Here we show an altered c-Fos expression in the brain of 2 month-old R6/1 mice following an operant conditioning task (Table 3). This alteration is not linked to changes of activity between the direct versus indirect pathway in the striatum, but could be rather associated with an alteration of PV+ IN activation (Table 4). Correlative analyses of c-Fos expression in several brain regions also reveal an early alteration of the functional neuronal network in R6/1 mice.

### Increased c-Fos expression following learning in 2 month-old motor pre-symptomatic HD mice

We first assessed the functional integrity of the different cellular populations of the striatum in R6/1 HD mice at 2 and 4 months by quantifying c-Fos expression produced by cognitive stimulation. Performing one single session of instrumental conditioning induced a significant increase of c-Fos expression in the striatum and other limbic regions as compared to home cage control condition, and this in all groups of mice including symptomatic transgenic mice. However, contrary to our expectation, the conditioning learning produced, in 2-month-old pre-symptomatic R6/1 mice as compared to age-matched WT littermates, a dramatic increase of c-Fos+ cell counts in the DMS and a moderate increase in the prefrontal cortex and CA1 of the hippocampus. Intriguingly, this “hyperactivity” went away at later symptomatic ages (i.e. 4- and 6-months) such that no genotype difference was observed at these ages.

Interestingly, the DMS hyperactivation in asymptomatic R6/1 mice was associated, first, with relatively preserved capacity for exploration of a novel environment (i.e. operant chamber) and a moderate but significant decrease in rewards obtained during the task. Secondly, the early striatal hyperactivity also was associated with a diminished c-Fos+ cell counts within PV interneuronal population within this region. Thirdly, the early striatal hyperactivity at this young age was also associated with an altered functional connectivity in R6/1 mice; the correlated activation during learning involved only restricted brain regions of the basal ganglia and the limbic circuit as compared to broad regions co-activated in age-matched WT littermates. Moreover, any correlative activity was found in home cage control condition in the mutant mice of this age, while wide network centered on the lateral amygdala was noticeable in WT littermates.

The reasons for these precocious changes of c-Fos expression in R6/1 mice are currently obscure. The observed phenomenon may be either a pathological process itself including changes of the molecular pathway leading to c-Fos activation, or adaptive and compensatory cellular responses to HD related changes or both. The illustration of the latter case comes from fMRI studies that revealed an increased cortical activity while preclinical HD patients were performing a memory task [28–30]. Our study demonstrated that the DMS under the cognitively challenging condition is capable of dramatic hyper-reactivity at early asymptomatic ages, ages at which we have recently shown an early functional and molecular synaptic alteration in these mice [31]. Therefore, it is tempting to speculate for a link between these observations.

### Disproportional vulnerability of indirect versus direct pathway MSNs was not found in R6/1 mice

These early and major changes of the DMS were not associated with differential activation patterns, in our R6/1 mice, of the two subtypes of MSNs. In other words, the similar ratio of indirect versus direct pathway MSNs were activated in both genotypes and ages studied. This observation is surprising because studies performed on both post-mortem HD brains and HD mice repeatedly suggested substantial and differential changes of biochemical and intrinsic cell property of indirect and direct pathway MSNs. In addition, one pharmacological study has also demonstrated that the D2R antagonism, in symptomatic R6/2 mice, attenuated response of immediate early genes including c-Fos, while the D1R agonism produced rather similar or enhanced striatal responses, suggesting clear differences in functional integrity between the direct and indirect pathway MSNs in HD mice [32]. However, our data seem to demonstrate that behavioral/physiological stimulation induced normal activation of striatal indirect and direct pathway MSNs in our HD mice.

Our data also confirm that both pathways were activated during nose-poke behaviors to be associated with food rewards [33] and provide “quantitative” information that two thirds of the recruited MSNs were those forming direct pathway in WT (and R6/1) mice against only one third forming indirect pathway. This pattern of activation could not be attributed to heterogeneous distribution of direct and indirect pathway MSNs in the specific area of the striatum studied here as equivalent numbers of both MSN subtypes were reported in the DMS in mice [34]. A physiological stimulation such as chronic wheel learning induced preferential activation of immediate early gene of direct pathway MSNs [35–36], while chronic stress and chronic appetitive stimuli and calorie restriction inducing increased locomotor activity and motivational state, increase equally immediate early gene expression of both pathways [37]. Therefore, the pattern of c-Fos activation of direct versus indirect pathway MSNs seems depending on the nature of physiological stimulation. Our data are in agreement with a model where both direct and indirect pathways are connected by interneuronal synaptic signaling and are not functionally segregated [38]. Importantly, this concurrent activity pattern is not altered in R6/1 mice even at motor symptomatic ages.

### Early and steady functional alterations of PV-expressing INs in R6/1 mice

PV expressing INs, representing less than 1% of striatal cells population, receive a direct excitatory cortical activation and exert a strong feed-forward inhibition to MSNs. These cells also are known to be more excitable than MSNs in physiological condition, while silent *in vitro* [15, 39–41]. Our observation in WT mice that approximately 30% of PV cells in the DMS express c-Fos is, therefore, coherent with their reported intense activity *in vivo* during cognitive tasks in rodents [15, 39–42]. This class of INs turned out to be severely dysfunctional in R6/1 mice, before any motor symptom manifestation. The diminished feed-forward inhibition of PV INs to MSNs may well be linked to the increased MSN activity observed in our HD mice at an early pre-symptomatic stage, but was not anymore associated with an increased c-Fos expression in MSNs at later age (4 months), suggesting further alterations of post-synaptic MSNs activity in symptomatic R6/1 mice.

While no consensus has been reached as to whether striatal interneuron populations also are impacted by HD mutation [8–9, 11, 43–44], *in vitro* electrophysiological studies did demonstrate their altered functional integrity in HD mice [10, 13]. Our data confirm and highlight severe and early dysfunctioning of PV INs in our HD mice.

### Changes of functional connectivity in R6/1 mice

While HD is classically associated with fatal neuronal death, accumulating evidence suggests that cellular dysfunction underlies HD symptoms. As a structural basis for the early alterations in neuronal function, several studies documented a significant reduction in the number of axonal fibers and synaptic proteins in HD, causing alterations in synaptic transmission [31, 45–47] and synaptic plasticity [48–55]. These synaptic functional changes have been directly linked to early cognitive and behavioral symptoms in different mouse models of HD [56–57].

Concomitantly, our HD mice also displayed altered network connectivity, which took place already at the very early pre-symptomatic stage in HD. In addition, such connectivity change also was observable while pre-symptomatic R6/1 mice are at rest in their home cages, similar to default mode network changes found in pre-symptomatic HD patients even in the absence of behavioral deficits [58–59]. This indicates and confirms that neural dysfunctions occur earlier than cell degeneration in HD [59].

## Conclusion

Here we report that significant changes of striatal activity *in vivo* were found in our HD mice at early ages at which no overt behavioral phenotypes are observed. The neuronal activity changes are the cortico-striatal hyperactivation, diminished activation of PV INs in addition to disorganization of functional brain networks. Dissecting the cellular and molecular changes arising at this young age may provide useful information for the identification of early biomarker as well as new therapeutic approaches aimed at delaying the disease onset.

## Supporting information

S1 Table ARRIVE check list. (PDF)

S2 Table Raw data. (XLSX)

## Abbreviations

AMG: Amygdala
CalR: Calretinin
ChAT: Acetylcholinesterase
D1R: D1 receptor
D2R: D2 receptor
DG: Dentate gyrus
DLS: Dorsolateral striatum
DMS: Dorsomedial striatum
HD: Huntington disease
IN: Interneuron
nNos: Neuronal nitric oxide synthase
PFC: Prefrontal cortex
PV: Parvalbumin
SEP: Septum
